# Roentgenoscopy of laser-induced projectile impact testing

**DOI:** 10.1107/S1600577524003898

**Published:** 2024-06-06

**Authors:** Xue Wang, Chunxia Yao, Bingbing Zhang, Dongsheng Zhang, Caijuan Shi, Ye Tao, Darui Sun

**Affiliations:** a Multi-Disciplinary Research Division Institute of High Energy Physics 19B Yuquan Road, Shijingshan District Beijing 100049 People’s Republic of China; b University of Chinese Academy of Sciences No. 1 Yanqihu East Rd, Huairou District Beijing101408 People’s Republic of China; University of Malaga, Spain

**Keywords:** laser-induced projectile impact testing, synchrotron radiation, dynamics, aerogel, dynamic imaging

## Abstract

Laser-induced projectile impact testing (LIPIT) based on synchrotron imaging is proposed and validated. This emerging high-velocity, high-strain microscale dynamic loading technique offers a unique perspective on the strain and energy dissipation behavior of materials subjected to high-speed microscale single-particle impacts. When combined with synchrotron radiation imaging techniques, LIPIT allows for *in situ* observation of particle infiltration. Two validation experiments were carried out, demonstrating the potential of LIPIT in the roentgenoscopy of the dynamic properties of various materials. With a spatial resolution of 10 µm and a temporal resolution of 33.4 µs, the system was successfully realized at the Beijing Synchrotron Radiation Facility 3W1 beamline. This innovative approach opens up new avenues for studying the dynamic properties of materials *in situ*.

## Abstract 2

Laser-induced projectile impact testing (LIPIT) based on X-ray white-light imaging is presented. This study confirms the potential of LIPIT based on synchrotron *in situ* imaging, which allows the recording of the trajectories of high-speed particles inside a material and the material’s impact response in real time.

## 1. Introduction

Testing under near-real conditions of, for example, temperature, humidity and radiation is essential for engineering applications of materials. The dynamics and energy dissipation behavior of materials under actual operating conditions can help us to fully understand the properties of materials. Impact testing is a widely used and highly controllable dynamic testing method that plays a vital role in fields ranging from powder blasting and cold spraying (Belloy *et al.*, 2000 ▸; Assadi *et al.*, 2016 ▸) to microscale additive manufacturing (Behera *et al.*, 2021 ▸), weapons development and aerospace (Redmann *et al.*, 2021 ▸; Marrs *et al.*, 2021 ▸). High strains and strain rates under impact are closely related to extreme properties of materials, such as nonlinear viscoelasticity, failure and explosion mitigation. In recent years it has been found that materials exhibit unexpected properties and kinetic behavior at high strain rates and small size scales (Jang & Greer, 2010 ▸; Peng *et al.*, 2022 ▸; Hyon *et al.*, 2018 ▸). The Laser-Induced Particle Impact Testing (LIPIT) platform is a dynamic loading tool suitable for investigating the impact behavior of micrometre-sized single particles of materials at high speeds. The LIPIT technique was first demonstrated by Lee *et al.* in 2010 as a characterization method that allows for quantitative high-strain rate impacts (Lee *et al.*, 2012 ▸). One of the main advantages of the technique is that it allows for precise quantification of the kinetic parameters of microparticles before, during and after impact. With a high-frame-rate camera, capturing the impact process at high temporal resolution (nanoseconds) and spatial resolution (submicrometre) is possible. LIPIT can provide highly safe and controllable loading of extreme high-strain impacts for studying the extreme kinetic behavior of materials. Therefore, it is widely used in studies of the kinetic properties of nano-lamellar, bulk and filamentary materials (Hyon *et al.*, 2021 ▸; Xie & Lee, 2020 ▸; Xie *et al.*, 2019 ▸; Veysset *et al.*, 2016 ▸; Fu *et al.*, 2014 ▸).

However, the current LIPIT methods have limitations. They are primarily used for *in situ* study of impact processes in visible light, which limits their potential. In the course of impacting, changes inside the material are not visible, which hampers studies of strain, energy dissipation and damage mechanisms. A perspective on the deformation inside the material and trajectories of the microparticles during *in situ* loading could provide richer observational data about the dynamic properties of materials under high strains and better explanations for the strain response mechanisms of materials.

*In situ* synchrotron X-ray imaging technology has the advantages of high penetration depth and high resolution. The main imaging modes include absorption, phase and tomography imaging, widely applied in additive manufacturing, batteries, propellants and so on (Lou *et al.*, 2021 ▸). For example, quantitative information revealed in the internal morphology and changes in the structure of energy materials during charging and discharging processes helps to understand their physicochemical mechanisms better (Takahashi *et al.*, 2016 ▸; Bak *et al.*, 2018 ▸); studying the mechanism and the law of the generation of melt pools and keyholes during additive manufacturing help improve the process parameters for better mechanical properties and fewer defects (Ioannidou *et al.*, 2022 ▸; Leung *et al.*, 2018 ▸; Zhao *et al.*, 2022 ▸); and analyzing the microevolution of the surface and interior of propellants during combustion elucidates the damage mechanism of the propellant (Luo *et al.*, 2023 ▸; Liu *et al.*, 2023 ▸). Therefore, *in situ* synchrotron X-ray imaging has become an essential research tool in many fields. On this basis, X-ray*in situ* imaging can fill the gap of the conventional LIPIT device and realize high temporal resolution and high spatial resolution perspective observation of the internal processes under high strain impact.

This paper presents a LIPIT based on X-ray white-light imaging at the Beijing Synchrotron Radiation Facility (BSRF) 3W1 beamline. The entire record of the microparticle infiltration process is obtained using two morphologies of aerogel samples as study objects. The protection of block aerogel against particle impact and the entire capture process of impacted particles by aerogel felts were observed. The energy dissipation characteristics of the block aerogel protection against particle impact and the kinetic behavior of flying particles decelerated and captured in aerogel felt were analyzed by recording the velocity and trajectory changes of the particles. This study confirms the potential of the LIPIT based on synchrotron *in situ* imaging and provides concrete evidence of its effectiveness. The LIPIT allows the trajectories of high-speed particles inside the material and the impact response of the material to be recorded in real time, making it an essential tool for a more comprehensive analysis of the impact dynamics behavior of microparticles and the microscale dynamic response of materials under high speed and strain.

## 2. Principle

**Figure 1:**
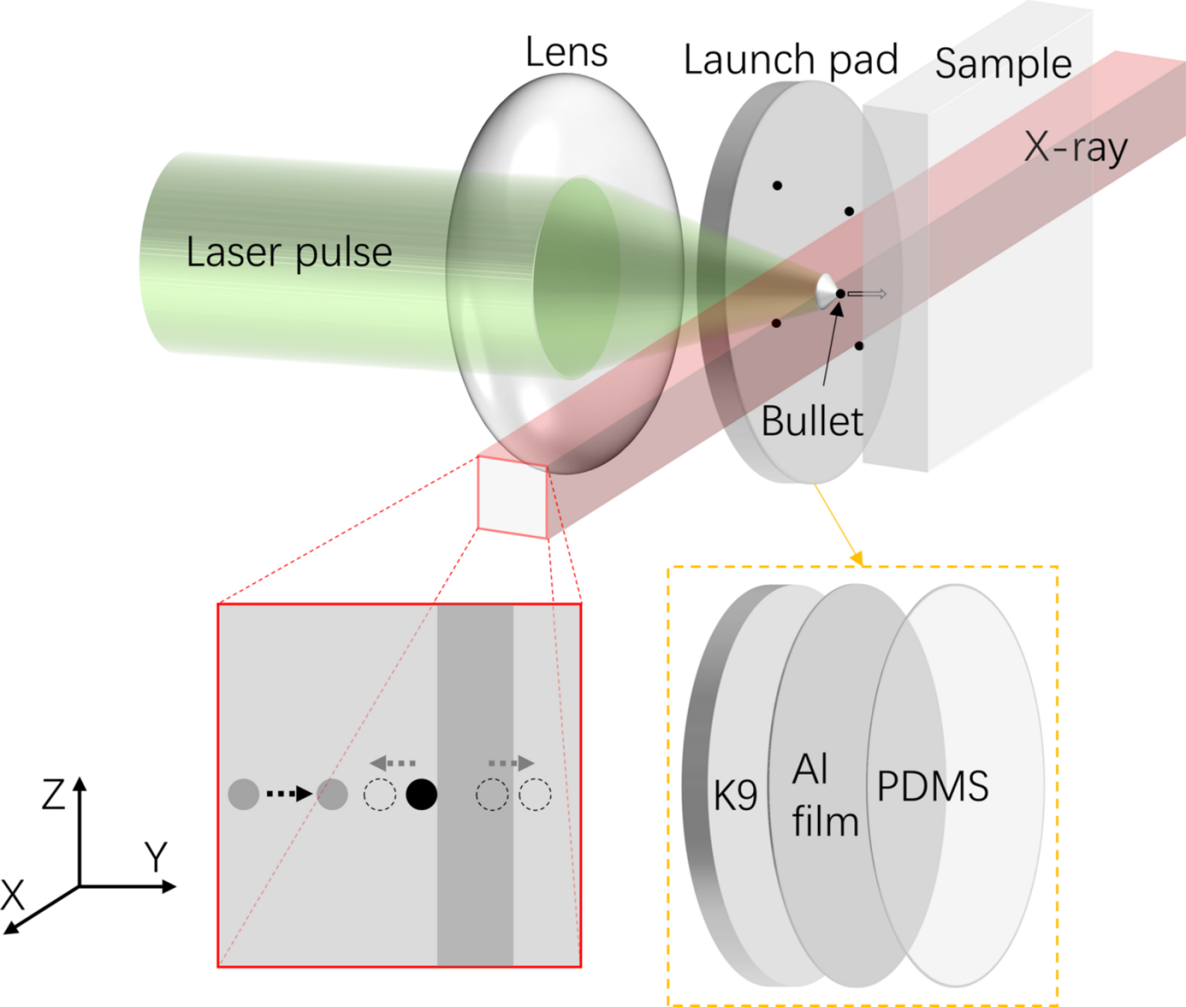
Schematic diagram of LIPIT based on *in situ* synchrotron X-ray imaging technology.

A schematic diagram of the LIPIT based on *in situ* synchrotron X-ray imaging technology is shown in Fig. 1 ▸, including a high power density laser, lens, launch pad and sample. The launch pad comprises a K9 glass substrate, an ablation layer (aluminium film) and a highly elastic film (polydi­methyl siloxane, PDMS). The lens focuses the laser, and then the aluminium film is ablated to generate plasma, rapidly expanding to drive the highly elastic film. Next, the bullet (microparticles) attached at the PDMS accelerates to a high initial velocity, and then the microparticles fly toward the sample at high speed. A rectangular X-ray beam parallel to the surface of the launch pad passes through a certain depth inside the sample. The process of particle acceleration, impact, rebound and intrusion is projected onto the detector. The system is a good combination of the high speed and high strain microscale impact characteristics of the LIPIT and the high penetration, spatial and temporal resolution of synchrotron radiation.

**Figure 2:**
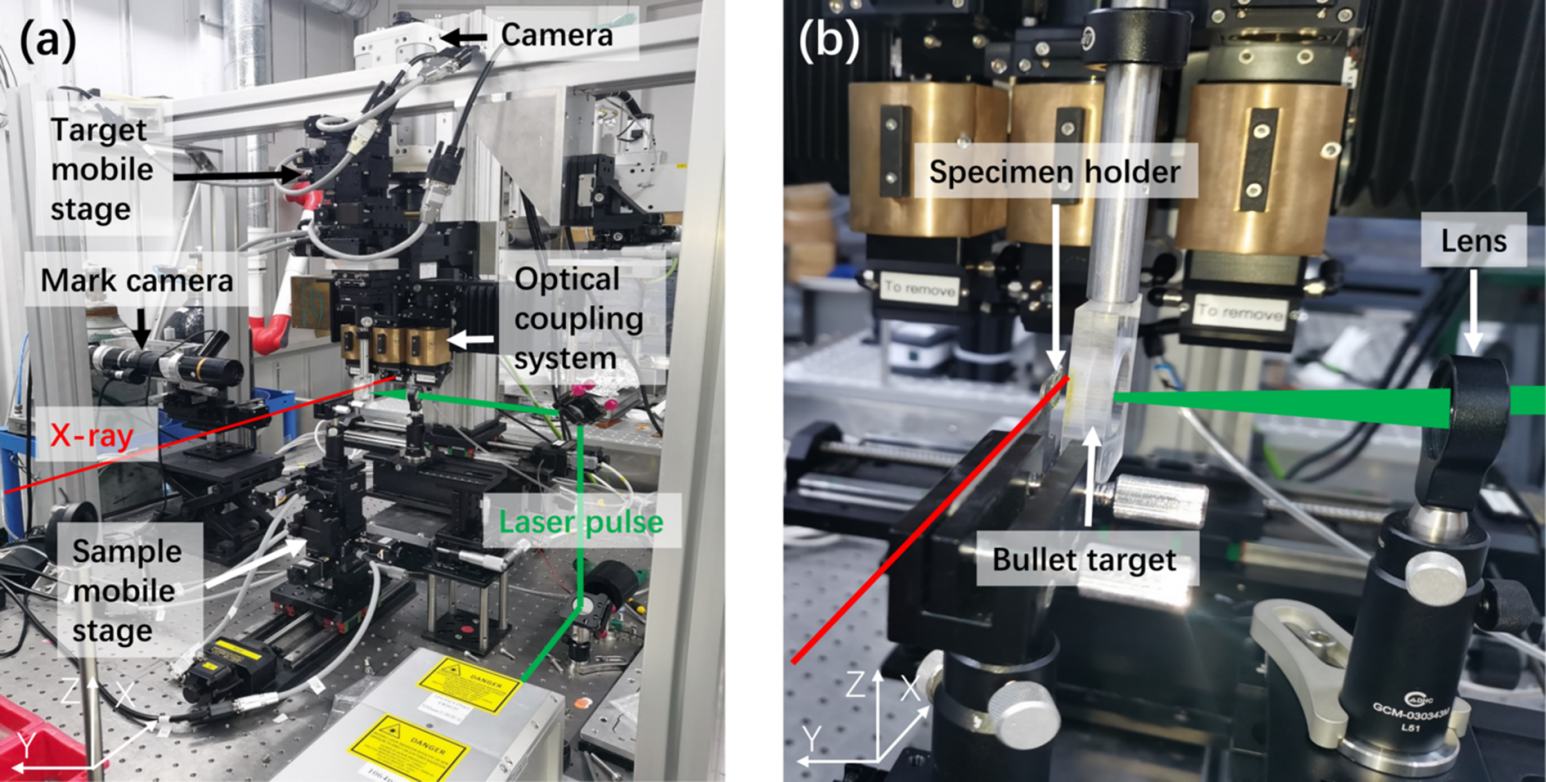
Diagram of the LIPIT experimental system based on synchrotron radiation.

In order to verify the feasibility and reliability of LIPIT based on synchrotron radiation imaging, a LIPIT experimental system was built at the BSRF 3W1 beamline, as shown in Fig. 2 ▸. The red line shows the direction of X-rays which is based on the superconducting wiggler insert. The synchrotron X-ray performance parameters are shown in Table 1 ▸. The laser wavelength of the LIPIT is 1064 nm (or a frequency-doubled 532 nm) emitted by an Nd:YAG laser. The laser is focused on the launch pad after two reflections through a lens with a focal length of 100 mm, and the aluminium film (tens of micrometres thick) absorbs the laser energy and instantaneously generates high-temperature and high-pressure plasma. The plasma rapidly expands to drive the deformation of the unabraded aluminium film and the high elasticity membrane (PDMS), pushing the attached microparticles (steel balls with a diameter of ∼300 µm) to accelerate and impact the sample. In the process, the PDMS acts as an insulator against the high temperature of the plasma that destroys the micro-particles. A visible-light camera (FASTCAM SA-Z; maximum frame rate 224 000 frames s^−1^) and multi-objective white X-ray beam imaging microscope (Optique Peter, X5) with a scintillator (200 µm-thick Ce:LuAG scintillator) was used to record the process. The spatial resolution of this imaging system was found to be 10 µm by imaging the test target (USAF1951). The temporal resolution of 33.4 µs is attained at a frame rate of 30000 frames s^−1^ (exposure time = 33.4 µs). The temporal resolution is limited by the flux intensity of the X-rays at 3W1. The study’s objective is to observe the change of the sample under impact. When impact occurs, the particles slow down considerably. The sample’s displacement is very small. The motion blur of the projectile does not affect the objectives. When it is time to study the moving particles, faster cameras, such as the KIRANA 7M, can help reduce motion blur.

Since high-dose X-ray irradiation can damage the samples, the exposure time of the X-rays during the experiment was controlled using a shutter. A DG645 Digital Pulse Delay Generator synchronizes the operating time of the laser, X-rays and camera. With minimal X-ray dose exposure, a complete record of the experimental process is ensured.

**Figure 3:**
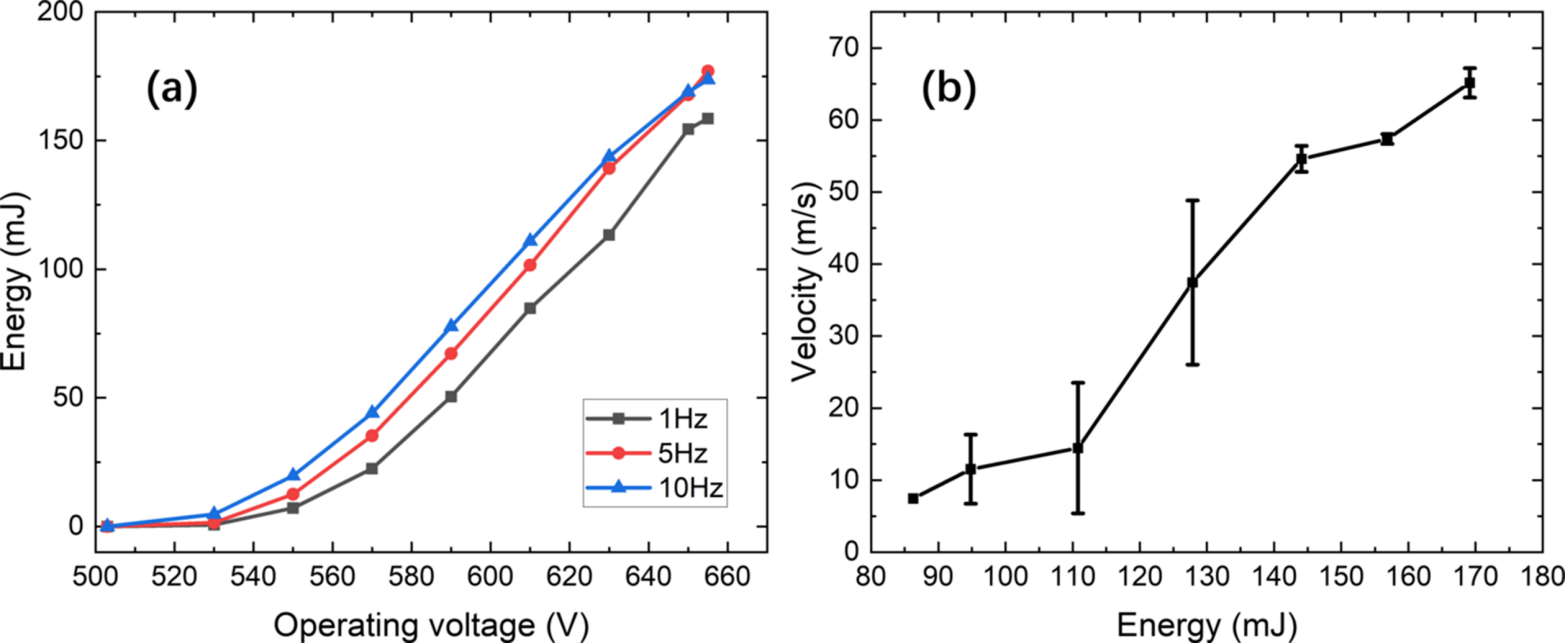
(*a*) Variation of the laser pulse energy with operating voltage. (*b*) Relationship between the emission velocity of the microparticles and laser pulse energy.

The velocity of the microparticles can be precisely and flexibly changed by adjusting the output power of the driving laser. Fig. 3 ▸(*a*) shows the variation of the laser output power with the operating voltage at repetition frequencies of 1 Hz, 5 Hz and 10 Hz. The pulse widths range from 7.15 to 24.70 ns. Fig. 3 ▸(*b*) shows the launching speed of the microparticles under different operating voltages. The power of the laser shows an S-shaped increase with operating voltage at different repetition frequencies. The high repetition frequency exhibits slightly higher output power at the same operating voltage. At each operating voltage, three microparticles were launched to measure their statistical velocity. The uniformity of the microparticle mass and the collimation of the microparticle with the focusing laser will play an essential role in the flexibility and controllability of the experiment. Since LIPIT is a quasi-energy-conserving process and the mass of the particles is non-negligible compared with the mass of the highly elastic film, the maximum particle velocity depends on the particle mass and the laser power. Higher velocities and smaller microscale regions can achieve a higher strain response. The highest velocity recorded using LIPIT is close to 4 km s^−1^ with silicon spheres with a diameter of 3.7 µm (Lee *et al.*, 2012 ▸). Therefore, LIPIT is uniquely vital in studying high-velocity microscale dynamical processes.

## 3. Result

An aerogel is a highly lightweight, porous solid material composed of nanocolloidal particles or polymer molecules. It exhibits exceptionally extreme material properties, such as a high specific surface area, a high strength-to-mass ratio, and an extremely low density and thermal conductivity. The main methods of aerogel preparation are supercritical drying and the emerging process of 3D printing aerogels (Zhao *et al.*, 2020 ▸; Aegerter *et al.*, 2011 ▸). Aerogels have a wide range of applications from aerospace to everyday civil applications (Gurav *et al.*, 2010 ▸), *e.g.* good building insulation, fuel storage media, catalyst carriers, Cherenkov detectors and blast impact protection materials.

**Figure 4:**
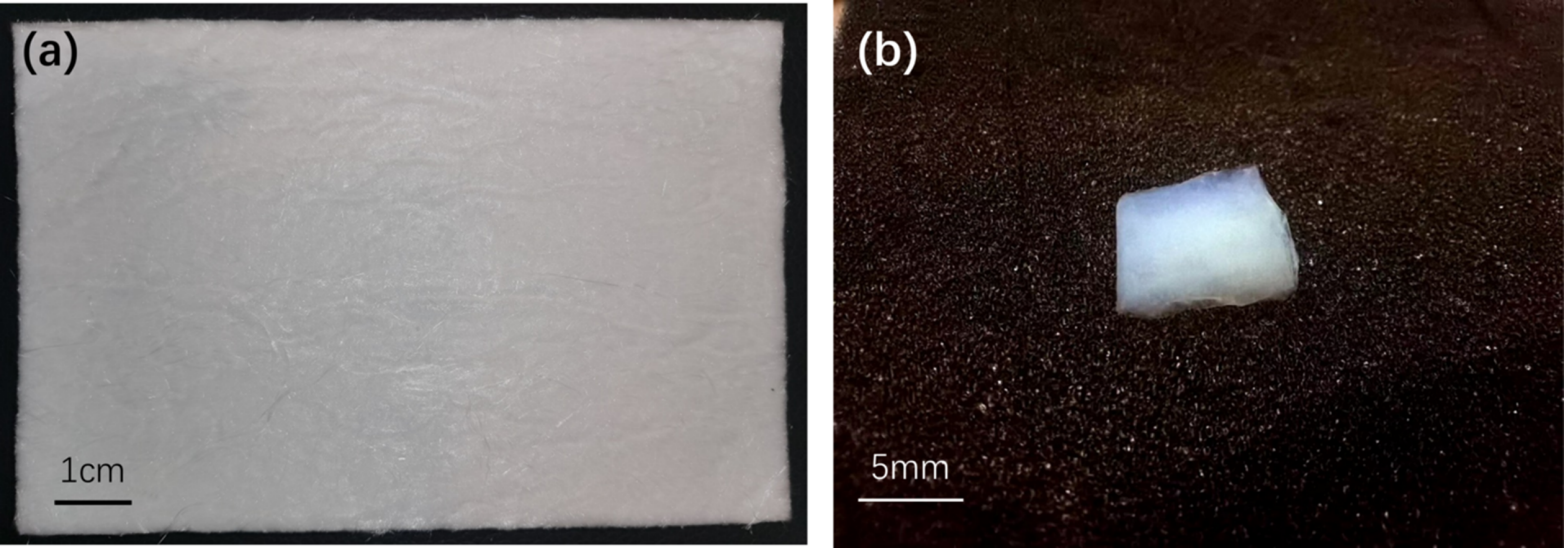
(*a*) Aerogel felt. (*b*) Block silica aerogel.

Two morphologies of aerogel, *i.e.* aerogel felt and bulk aerogel, as shown in Figs. 4 ▸(*a*) and 4(*b*), were used to investigate two processes: flying particles capture and impact protection, respectively. Aerogel felts consist of aerogel particles and inorganic fibers, and have a thickness of 5 mm and a density of 180 ± 30 kg m^−3^. Bulk aerogels are composed of crosslinked reticulated silica nanowires. They have a purity of greater than 99%, density of 40–150 kg m^−3^ and pore sizes of between 20 and 100 nm.

### 3.1. Aerogel felts capture impact particles

**Figure 5:**
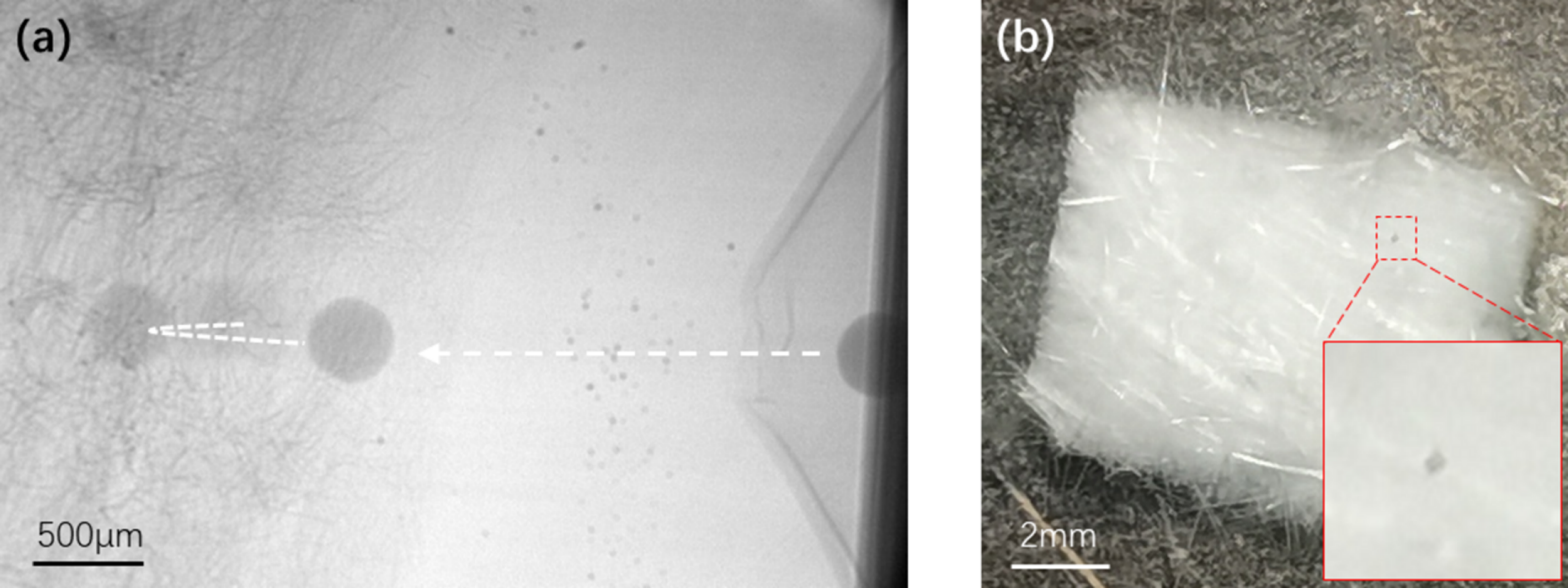
(*a*) Trajectory of microparticles captured by the aerogel felt. The particle speed was 65 m s^−1^. (*b*) Microparticles inside aerogel felt after the experiment.

As shown in Fig. 5 ▸(*a*), the motion trajectories of microparticles impacting the aerogel felt were captured by high-speed camera. The operating voltage was 650 V during the experiment so that the microparticles could travel at up to the maximum speed allowed under the experimental conditions. The frame rate of the camera was 30000 frames s^−1^, and the diameter of the microparticles was 300 µm. The velocity of the microparticles reached 65 m s^−1^. The contour of the bulging PDMS can be seen on the right of the figure, as well as the contour of the aerogel felt and the inorganic fibers on the left of the figure. The microparticle travels a short distance before impacting the aerogel felt, and then decelerates due to the drag force as it moves inside the aerogel. As the aerogel felt has low elasticity, the particles are subjected to a rebound force of the compressed aerogel felt and slightly rebound after entering maximum depth. Finally, the movement of the microparticle stops and the microparticle is captured by the aerogel felt. The aerogel felt was peeled off after the experiment. The red dashed box in Fig. 5 ▸(*b*) indicates the captured microparticles inside the felt. The results show that the aerogel felt can quickly reduce the velocity of impact microparticles and capture flying particles.

### 3.2. Aerogel impact testing

**Figure 6:**
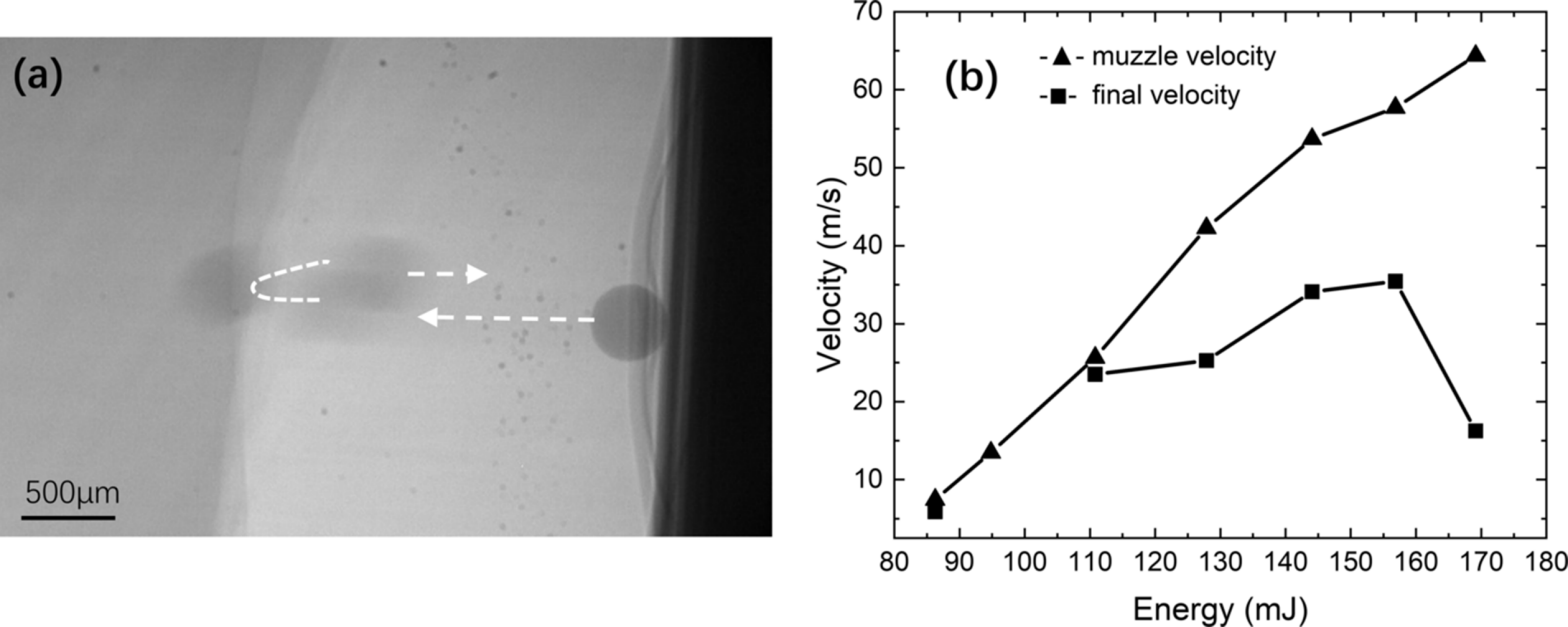
(*a*) Trajectory of microparticles impacting a block aerogel. The speed of the particle was from 9.6 m s^−1^ to 65 m s^−1^. (*b*) Variation of the initial velocity and rebound velocity of particles with laser pulse energy.

The motion trajectory of microparticles impacting a block aerogel is schematically shown in Fig. 6 ▸(*a*). The initial velocity of the microparticles gradually increases with the operating voltage from 9.6 m s^−1^ to 65 m s^−1^. Due to the rigid characteristics of the bulk aerogel, the microparticles rebound back after they reach the aerogel’s surface. The kinetic energy loss of the microparticles upon impacting the aerogel can be calculated by recording the rebound velocity: 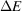 = 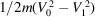, where *m* is the mass of the microparticle, 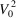 denotes the initial velocity, and 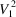 denotes the rebound velocity. The velocity of the microparticles at different moments can be obtained by analyzing the position change of the particles via the high-speed camera. Fig. 6 ▸(*b*) shows the initial and rebound velocity of the microparticles with different operating voltages, where the black solid triangles indicate the initial velocity and the black solid squares indicate the rebound velocity. The result indicates that the initial velocity increases rapidly with laser pulse energy, and the rebound velocity shows a tendency to slowly increase and then decrease. Under impact loading, the internal stress of the aerogel rises rapidly, and the nanoporous structure is gradually destroyed. The fiber breaks, the specific surface area decreases and the pore volume shrinks, which causes a loss of energy. The aerogel shows a strain-rate hardening and increased energy absorption under dynamic compression because of the internal gas on the hole. As a result, the aerogel shows good anti-impact performance against high-speed impacts on the micrometre scale.

## 4. Conclusions and outlook

In this article LIPIT based on synchrotron radiation is introduced. The device combines the high spatial and temporal resolution *in situ* perspective capability of synchrotron radiation and the high speed and high strain rate micro-region impact characteristics of LIPIT.

The whole process of the impact microparticles captured by aerogel felt, and the microparticles impacting a block aerogel with different velocities is recorded by roentgenoscopy. The result shows that combining the two techniques can provide a powerful tool for studying the dynamic properties of materials *in situ*. A perspective observation of the microparticle impact was analyzed, including the trajectories of microparticles inside the sample, the changes in velocity, and morphological features on the surface and inside of the material. However, the imaging performance in this experiment is limited by the energy and flux of the 3W1 beamline. Temporal resolution of ∼30 µs and spatial resolution of 10 µm are achieved. With the application of higher-energy synchrotron radiation sources, the range of materials that the device can investigate will be significantly expanded, such as all kinds of synthetic materials with eminent mechanical performances. At the same time, the spatial and temporal resolution of the imaging will be improved to the nanoscale with nanosecond time resolution.

## Figures and Tables

**Table 1:** Parameters of the 3W1 beamline Beijing Synchrotron Radiation Facility

Insert	Magnetic field strength	Energy range	Energy resolution	Characteristic energy	Flux	Spot size
Superconducting wiggler	2.6 T	White light	−0.16% @ 50 keV	10.2 keV	2 × 10^14^ photons s^−1^ @ white	9.37 mm × 2.47 mm

## References

[bb1] Aegerter, M. A., Leventis, N. & Koebel, M. M. (2011). *Aerogels Handbook.* Springer Science & Business Media.

[bb2] Assadi, H., Kreye, H., Gärtner, F. & Klassen, T. (2016). *Acta Mater.* **116**, 382–407.

[bb3] Bak, S.-M., Shadike, Z., Lin, R., Yu, X. & Yang, X.-Q. (2018). *NPG Asia Mater.* **10**, 563–580.

[bb4] Behera, D., Chizari, S., Shaw, L. A., Porter, M., Hensleigh, R., Xu, Z., Zheng, X., Connolly, L. G., Roy, N. K., Panas, R. M., Saha, S. K., Zheng, X., Hopkins, J. B., Chen, S.-C. & Cullinan, M. A. (2021). *Precis. Eng.* **68**, 197–205.

[bb5] Belloy, E., Thurre, S., Walckiers, E., Sayah, A. & Gijs, M. A. M. (2000). *Sens. Actuators A Phys.* **84**, 330–337.

[bb6] Fu, R. R., Weiss, B. P., Lima, E. A., Harrison, R. J., Bai, X. N., Desch, S. J., Ebel, D. S., Suavet, C., Wang, H., Glenn, D., Le Sage, D., Kasama, T., Walsworth, R. L. & Kuan, A. T. (2014). *Science*, **346**, 1089–1092. 10.1126/science.1258022 25394792

[bb7] Gurav, J. L., Jung, I.-K., Park, H.-H., Kang, E. S. & Nadargi, D. Y. (2010). *J. Nanomater.* **2010**, 1–11.

[bb8] Hyon, J., Lawal, O., Fried, O., Thevamaran, R., Yazdi, S., Zhou, M., Veysset, D., Kooi, S. E., Jiao, Y., Hsiao, M.-S., Streit, J., Vaia, R. A. & Thomas, E. L. (2018). *Mater. Today*, **21**, 817–824.

[bb9] Hyon, J., Lawal, O., Thevamaran, R., Song, Y. E. & Thomas, E. L. (2021). *Adv. Sci.* **8**, 2003142. 10.1002/advs.202003142 PMC7967058 33747728

[bb10] Ioannidou, C., König, H., Semjatov, N., Ackelid, U., Staron, P., Körner, C., Hedström, P. & Lindwall, G. (2022). *Mater. Des.* **219**, 110790.

[bb11] Jang, D. & Greer, J. R. (2010). *Nat. Mater.* **9**, 215–219. 10.1038/nmat2622 20139966

[bb12] Lee, J. H., Veysset, D., Singer, J. P., Retsch, M., Saini, G., Pezeril, T., Nelson, K. A. & Thomas, E. L. (2012). *Nat. Commun.* **3**, 1164. 10.1038/ncomms2166 23132014

[bb13] Leung, C. L. A., Marussi, S., Atwood, R. C., Towrie, M., Withers, P. J. & Lee, P. D. (2018). *Nat. Commun.* **9**, 1355. 10.1038/s41467-018-03734-7 PMC5893568 29636443

[bb14] Liu, Y., Qian, W., Wang, L., Xue, Y., Hou, C. & Wu, S. (2023). *Mater. Sci. Eng. A*, **882**, 145451.

[bb15] Lou, S., Zhang, F., Wang, H. & Wang, J. (2021). *Advanced X-ray Imaging of Electrochemical Energy Materials and Devices*, edited by J. Wang, pp. 1–25. Singapore: Springer Singapore.

[bb16] Luo, H., Lou, Y., He, K. & Jiang, Z. (2023). *Combust. Flame*, **249**, 112609.

[bb17] Marrs, F. W., Manner, V. W., Burch, A. C., Yeager, J. D., Brown, G. W., Kay, L. M., Buckley, R. T., Anderson-Cook, C. M. & Cawkwell, M. J. (2021). *Ind. Eng. Chem. Res.* **60**, 5024–5033.

[bb18] Peng, Y., Luo, G., Hu, Y. & Xiong, D. (2022). *Composites B*, **235**, 109763.

[bb19] Redmann, A., Montoya-Ospina, M. C., Karl, R., Rudolph, N. & Osswald, T. A. (2021). *Composites C*, **5**, 100136.

[bb20] Takahashi, H., Okazaki, R., Ishiwata, S., Taniguchi, H., Okutani, A., Hagiwara, M. & Terasaki, I. (2016). *Nat. Commun.* **7**, 12732. 10.1038/ncomms12732 PMC5025859 27597055

[bb21] Veysset, D., Hsieh, A. J., Kooi, S., Maznev, A. A., Masser, K. A. & Nelson, K. A. (2016). *Sci. Rep.* **6**, 25577. 10.1038/srep25577 PMC4860635 27156501

[bb22] Xie, W. & Lee, J.-H. (2020). *Macromolecules*, **53**, 1701–1705.

[bb23] Xie, W., Zhang, R., Headrick, R. J., Taylor, L. W., Kooi, S., Pasquali, M., Müftü, S. & Lee, J. H. (2019). *Nano Lett.* **19**, 3519–3526. 10.1021/acs.nanolett.9b00350 31084030

[bb24] Zhao, C., Shi, B., Chen, S., Du, D., Sun, T., Simonds, B. J., Fezzaa, K. & Rollett, A. D. (2022). *Rev. Mod. Phys.* **94**, 045002.

[bb25] Zhao, S., Siqueira, G., Drdova, S., Norris, D., Ubert, C., Bonnin, A., Galmarini, S., Ganobjak, M., Pan, Z., Brunner, S., Nyström, G., Wang, J., Koebel, M. M. & Malfait, W. J. (2020). *Nature*, **584**, 387–392. 10.1038/s41586-020-2594-0 32814885

